# Opposite Influence of Perceptual Memory on Initial and Prolonged Perception of Sensory Ambiguity

**DOI:** 10.1371/journal.pone.0030595

**Published:** 2012-01-25

**Authors:** Maartje Cathelijne de Jong, Tomas Knapen, Raymond van Ee

**Affiliations:** 1 Physics of Man, Helmholtz Institute, Utrecht University, Utrecht, The Netherlands; 2 Brain & Cognition Group, University of Amsterdam, Amsterdam, The Netherlands; 3 Department of Brain, Body & Behavior, Philips Research Laboratories, Eindhoven, The Netherlands; 4 Laboratory of Experimental Psychology, University of Leuven, Leuven, Belgium; 5 Department of Biophysics, Donders Institute, Radboud University, Nijmegen, The Netherlands; CNRS - Université Claude Bernard Lyon 1, France

## Abstract

Observers continually make unconscious inferences about the state of the world based on ambiguous sensory information. This process of perceptual decision-making may be optimized by learning from experience. We investigated the influence of previous perceptual experience on the interpretation of ambiguous visual information. Observers were pre-exposed to a perceptually stabilized sequence of an ambiguous structure-from-motion stimulus by means of intermittent presentation. At the subsequent re-appearance of the same ambiguous stimulus perception was initially biased toward the previously stabilized perceptual interpretation. However, prolonged viewing revealed a bias toward the alternative perceptual interpretation. The prevalence of the alternative percept during ongoing viewing was largely due to increased durations of this percept, as there was no reliable decrease in the durations of the pre-exposed percept. Moreover, the duration of the alternative percept was modulated by the specific characteristics of the pre-exposure, whereas the durations of the pre-exposed percept were not. The increase in duration of the alternative percept was larger when the pre-exposure had lasted longer and was larger after ambiguous pre-exposure than after unambiguous pre-exposure. Using a binocular rivalry stimulus we found analogous perceptual biases, while pre-exposure did not affect eye-bias. We conclude that previously perceived interpretations dominate at the onset of ambiguous sensory information, whereas alternative interpretations dominate prolonged viewing. Thus, at first instance ambiguous information seems to be judged using familiar percepts, while re-evaluation later on allows for alternative interpretations.

## Introduction

**Figure 1:**
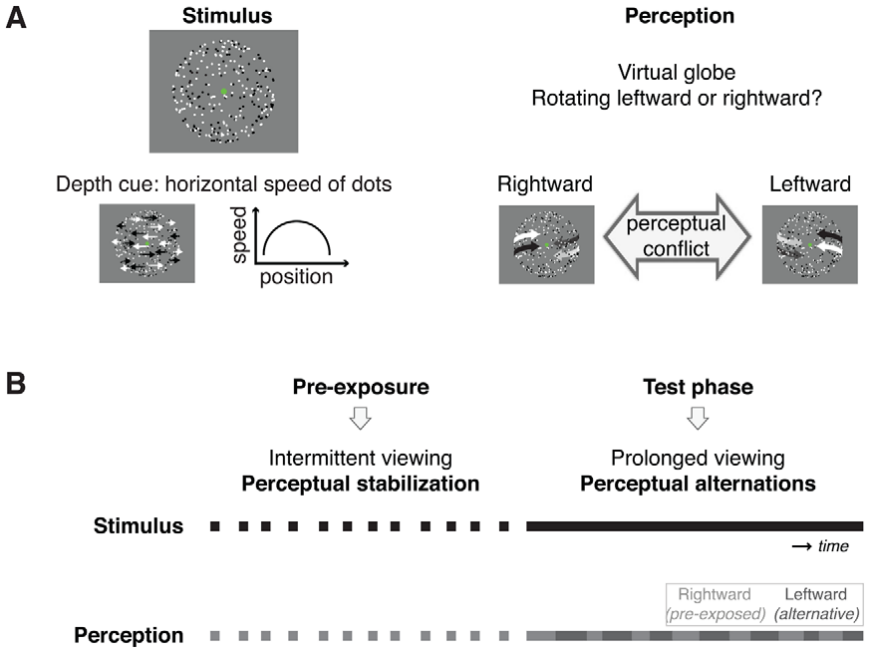
Stimulus and paradigm. **A**) The stimulus consisted of black and white leftward and rightward moving dots placed such that they represented points on the surface of a virtual globe. Depth was signaled by the sinusoidal speed profile of the dots, i.e. the dots moved faster as they were closer to the vertical meridian of the globe, thereby creating the illusion of a 3-dimensional globe in depth. The virtual globe was perceived rotating around its vertical axis, but the direction of the rotation was ambiguous: either the rightward or the leftward moving surface was perceived in front of the other surface. **B**) A trial started with an intermittent presentation period of variable duration (up to 4.3 minutes) during which the ambiguous globe perceptually stabilized. Subsequently, the ambiguous globe was presented continuously for a prolonged duration (up to 10 minutes). During this period perceptual alternations occurred every few seconds. The stabilized percept is referred to as the ‘pre-exposed’ percept throughout this manuscript. We investigated the effect of the pre-exposure on the durations of the pre-exposed and alternative percept, during continuous test period.

The visual input registered by our eyes is inherently ambiguous. To maintain a stable perceptual representation of the state of the world the brain has to make inferences. This means that observers continually, yet unconsciously, make perceptual choices based on ambiguous sensory information [1]. It is easily envisaged how such perceptual decision-making mechanisms may shape their performance by learning from experience [2]–[4]. In this study we investigated how our current perceptual interpretation of the outside world is influenced by previous perception. In order to dissociate prior *perception* from prior *stimulation* we used ambiguous visual input, i.e. stimuli that allow for several, mutually exclusive (‘rivalrous’), perceptual interpretations (example in fig. 1A). Under these conditions of ongoing ambiguity in the visual input we tested whether perceptual decisions from the recent past influence the detailed time-course of current perceptual decisions.

Visual input is generally associated with a definite perceptual state, even when the input is ambiguous. At the onset of an ambiguous stimulus only one of the possible perceptual interpretations is perceived (‘rivalry at onset’). Subsequently, a process of continuous perceptual alternations between the different interpretations sets in (‘ongoing rivalry’). Although these two aspects of rivalry are believed to involve the same neural populations, the processes of perceptual decision-making exhibit several differences. For example, the frequency of perceptual alternations is much lower when short presentations of an ambiguous stimulus are interleaved with blank intervals than when a single, longer-lasting, presentation of the stimulus is viewed continuously [5]. An intermittent paradigm can be thought of as the repeated occurrence of rivalry at onset, while a continuous paradigm reflects the mechanisms of ongoing rivalry. Other differences between rivalry at onset and ongoing rivalry concern the influence of perceptual biases [6], [7] and the influence of attention [8], [9].

The slow frequency of perceptual alternations during intermittent viewing is often referred to as ‘perceptual stabilization’ and is argued to reflect perceptual memory [10]–[12]. Here we utilized this phenomenon to build-up minutes-long perceptual experience with only one of the interpretations of an ambiguous stimulus, while the other perceptual interpretation was suppressed. This enabled us to investigate the influence of biased perceptual experience on current perception of ambiguous visual input. The buildup of biased perceptual experience would not have been possible using continuous presentation of an ambiguous stimulus, because in such a paradigm ongoing perceptual alternations occur. Perceiving such alternations can result in percept-invariant modulations of perception, for example an increase or decrease in perceptual alternation-rate (e.g. [13]–[15]), but it does not reveal percept-specific effects of perceptual experience. An alternative method to bias perceptual experience is to use pre-exposure to an unambiguous stimulus. However, we preferred ambiguous pre-exposure, because the features used to bias an unambiguous stimulus may induce feature-specific neuronal adaptation independent of their intended perceptual effect. In the case of perceptually biased, i.e. stabilized, ambiguous pre-exposure any percept-specific ‘memory’ or adaptation is related to the perceptual interpretation of the information and not to an imbalance in stimulation.

**Figure 2:**
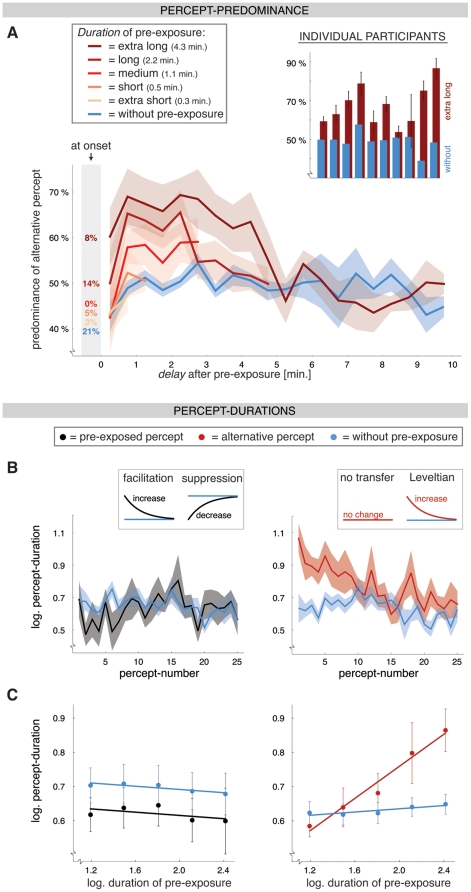
Results of Experiment 1: Ambiguous pre-exposure. **A**) On the left: Predominance of the alternative percept at the onset of the test phase (i.e. identity of the first percept; see numbers in grey shading) and during subsequent ongoing rivalry (±SEM; width of time-bins is 30 sec.) in five conditions with increasing duration of the pre-exposure (from *yellow* to *dark red*). The *blue* line reflects the averaged baseline measure (without pre-exposure) for the 5 different durations of pre-exposure (statistics reported in the text were done on the individual baseline measures). During continuous viewing the predominance of the alternative percept was larger after longer pre-exposure durations and decreased over time. Such an effect was not present at the onset of the test phase. On the right: Predominance of the alternative percept for individual participants after a pre-exposure period lasting 4.3 minutes. Here, the predominance was calculated over a time-window of 0.5 to 3.5 minutes after pre-exposure. Within this time-window the group-data for this condition significantly differed from the baseline measure. **B**) The average duration (±SEM) of the first to the 25^th^ percept without pre-exposure (*blue*) and after 4.3 minutes of pre-exposure (pre-exposed percept in *black*, left graph; alternative percept in *red*, right graph). The duration of the pre-exposed percept was not increased, even while this percept was facilitated in the sense that it was likely to occur at stimulus onset. The results for the pre-exposed percept resemble the suppression hypothesis (proposing a ‘fatigue-like’ effect) more than the facilitation hypothesis (proposing a ‘memory-like’ effect) (see inset in left graph). Although the alternative percept was not seen during pre-exposure, its duration shows a clear increase after pre-exposure, which might relate to Levelt's 2^nd^ proposition (Levelt, 1967). **C**) The average duration (±SEM) of the percepts that occurred within 1.5 minutes after the pre-exposure (pre-exposed percept in *black*, left graph; alternative percept in *red*, right graph) or within the first 1.5 minutes of the condition without pre-exposure (*blue*). Data are shown for five different durations of the pre-exposure. The duration of the alternative percept increased when the duration of the pre-exposure increased, whereas the duration of the pre-exposed percept remained unchanged.

We investigated the influence of minutes-long, perceptually stabilized, ambiguous pre-exposure on subsequent continuous perception of the same stimulus. Earlier studies have investigated the perceptual dynamics within a period of intermittent presentation (e.g. [12], [16]) or reported the first couple of seconds/percepts after the onset of rivalry [17], [18], but such short presentations of the stimulus mostly reflect the dynamics of rivalry at onset. A detailed analysis of the durations of the two percepts during ongoing rivalry can reveal the intimate properties of prolonged ambiguous perception. In line with the phenomenon of perceptual stabilization, which has been attributed to perceptual memory [10]–[12], we may expect a facilitation of the pre-exposed percept during ongoing rivalry, for example reflected in an increase in the average duration of the pre-exposed percept (facilitation hypothesis, fig. 2B).

Previous studies into *rivalry at onset* have reported either facilitation or suppression of the pre-exposed percept. Facilitation has been found particularly after ambiguous or faint/brief unambiguous pre-exposure, or with long intervals between the pre-exposure and the test stimulus [10], [11], [15], [16], [19]–[22]. Suppression of the pre-exposed percept, reflected in the tendency to see the alternative percept, is common with short intervals between the ambiguous pre-exposure and the test stimulus (e.g. [16]) or after strong unambiguous pre-exposure [23]–[26]. It has been attributed to ‘adaptation’, ‘satiation’ or ‘neural fatigue’ (e.g. [23]–[28]). Accordingly, an alternative hypothesis regarding our paradigm is that the average duration of the pre-exposed percept is decreased during ongoing rivalry, rather than increased, after ambiguous pre-exposure (suppression hypothesis, fig, 2B). We expect the duration of the alternative percept to be unaffected by pre-exposure, because this percept is not seen during the pre-exposure (‘no transfer’-hypothesis, fig. 2B). However, manipulations of one of the percepts can affect the duration of the opposite percept (second proposition of Levelt in [29], see also [30]), thus we should consider the possibility that the effect of pre-exposure transfers to the alternative percept (Leveltian hypothesis, fig. 2B).

Our results indicate that the pre-exposed percept was facilitated during rivalry at onset, but was not much affected during ongoing rivalry. Interestingly, the duration of the alternative percept, i.e. the percept that was suppressed during intermittent pre-exposure, increased during subsequent ongoing rivalry, supporting the Leveltian hypothesis (illustrated in fig. 2B). This effect occurred similarly for ambiguous structure-from-motion and binocular rivalry. During binocular rivalry the eye-bias was not affected by pre-exposure. In additional experiments we elaborate on the effects of specific characteristics of the pre-exposure, such as the comparison between ambiguous and unambiguous pre-exposure.

## Methods

### Participants

The number of participants was 10, 6, 13, and 6 for Experiments 1, 2, 3, and 4, respectively. Seven participants participated in more than 1 experiment. The remaining 18 participated only in 1 experiment. Most participants (20 out of 25) had no experience with psychophysical experiments. Participants who reported particular difficulty in perceiving the three-dimensional structure of the stimulus or differentiating the two possible percepts were excluded (8 out of 33). All participants gave verbal informed consent before participation and had normal or corrected to normal vision. All experiments were conducted in agreement with (not specifically approved by) the ethics and safety guidelines of the Science Faculty of Utrecht University.

### Experiment 1: Ambiguous pre-exposure

#### Stimulus and task

We used a structure-from-motion stimulus [31], [32] consisting of 450 leftward or rightward moving dots (each 0.077° in diameter). The dots represented random points on the surface of a virtual globe (5.0° in diameter). The globe rotated around its vertical axis with a period of 7.8 seconds. Stimuli were created using custom software and presented in the center of a gray computer-screen (75 Hz LaCie monitor, 1600×1200 pixels, a gamma shaped luminance correction was applied). The direction of rotation was ambiguous (leftward or rightward), because no depth cues differentiated the rightward moving surface from the leftward moving surface (fig. 1A). Observers alternately perceived either of two possible percepts for several seconds at a time. Participants were instructed to maintain strict fixation on a static green dot (0.18° in diameter) placed in the center of the globe. Head movements were constrained using a chin-rest. Participants indicated the direction of motion of the surface perceived to be in front by holding down one of two corresponding buttons on a keyboard, and releasing the buttons when the stimulus disappeared or when they could not differentiate the front from the back surface. During the intermittent presentations the participants were required to respond to every single presentation of the stimulus. Without explaining why, participants were informed that the rotation directions they were going to see were unpredictable and that their percepts were never ‘incorrect’. Upon debriefing afterwards most participants reported that they had been unaware of the perceptual ambiguity of the stimulus.

#### Procedure

Each trial consisted of two phases. In the pre-exposure phase the ambiguously rotating globe was presented intermittently to stabilize perception and build up ‘experience’ with one of the two possible percepts (duration of one presentation of the globe: 720 ms; duration of intervening blank periods: random value between 800 and 1200 ms). In the following test phase the ambiguously rotating globe was presented continuously to test the effect of the pre-exposure on prolonged viewing (fig. 1B). There were five conditions with distinct pre-exposure durations, being 16, 31, 64, 130, and 260 seconds (which is 0.3, 0.5, 1.1, 2.2, and 4.3 minutes, respectively). The corresponding durations of the test phase were 1.2, 1.7, 2.7, 5.0, and 10.1 minutes, respectively. The durations of the test phase were based on pilot experiments. These pilot experiments revealed no cyclic or late effects of pre-exposure after the effect seen in the beginning of the test phase. Two baseline measurements were added that lacked the pre-exposure phase (duration of test phase: 5.0 and 10.1 minutes; data were analyzed in conjunction). Most participants completed 4 trials per condition. Some participants completed fewer trials due to reduced availability of the participant or because of technical issues (on average 3.9 trials were completed). Of the completed trials a total of 4.0% was excluded from the analysis. Inclusion criteria for trials were: 1) during the pre-exposure phase one percept should be seen at least three times more often than the other percept, i.e. there should have been proper perceptual stabilization, and 2) during the pre-exposure as well as the test phase the subject should have reported either one of the two possible percepts in at least 75% of the time that the stimulus was displayed (subjects refrained from responding when they could not distinctly identify the rotation direction of the globe).

### Experiment 2: Unambiguous pre-exposure

**Figure 3:**
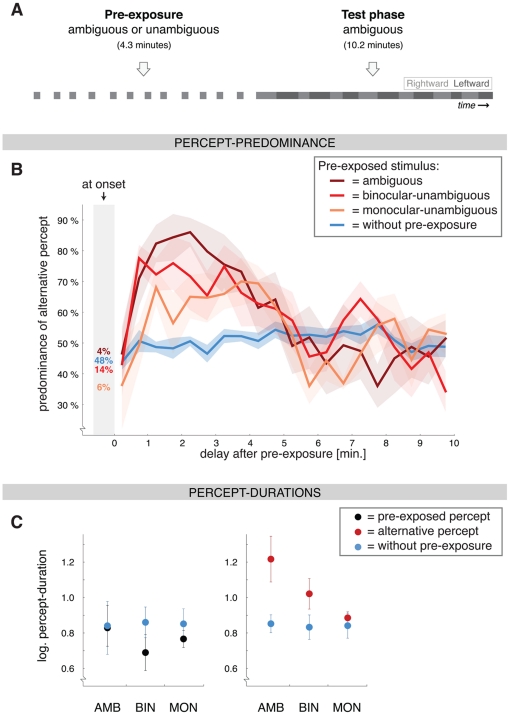
Paradigm and results of Experiment 2: Unambiguous pre-exposure. **A**) The paradigm. The pre-exposure period had a fixed duration (4.3 minutes) and contained either an ambiguous globe, a globe disambiguated using binocular depth-cues (disparity) or a globe disambiguated using monocular depth-cues (contrast- and size-imbalance). The subsequent test period always contained an ambiguous globe. **B**) The predominance of the alternative percept at the onset of the test phase (numbers in grey shading) and during subsequent ongoing rivalry (±SEM; bin-width: 30 sec.) in the condition without-pre-exposure (*blue*; averaged baseline measure) and after ambiguous (*dark red*), binocular-unambiguous (*red*) and monocular-unambiguous (*orange*) pre-exposure. After pre-exposure the predominance of the alternative percept was increased during continuous viewing (but not at onset) in all 3 conditions. This increase was successively larger for the monocular-unambiguous, binocular-unambiguous and ambiguous condition. **C**) The average duration (±SEM) of the percepts that occurred between 0.5 and 4.5 minutes after pre-exposure (pre-exposed percept in *black*, left graph; alternative percept in *red*, right graph; no pre-exposure in *blue*). The increase in the duration of the alternative percept was successively larger when the pre-exposed stimulus was monocular-unambiguous (MON), binocular-unambiguous (BIN) or ambiguous (AMB). The slight decrease in the duration of the pre-exposed percept did not significantly differ between the 3 conditions.

The pre-exposure phase of each trial (lasting 260 seconds/4.3 minutes) contained either an ambiguous, a ‘monocular-unambiguous’ or a ‘binocular-unambiguous’ globe, while the test phase (lasting 10.1 minutes) always contained an ambiguous globe (fig. 3A). The unambiguous globes were identical to the ambiguous globe, with the exception that cues were added to indicate an ordering in depth of the leftward and rightward moving dots. For the binocular-unambiguous globes we used *disparity*, a binocular depth cue. With a mirror stereoscope two slightly different images were presented to the two eyes, mimicking the different viewing angles that the two eyes would have on a globe in depth. The monocular-unambiguous globes were viewed with both eyes, but contained only monocular depth cues: 1) *contrast imbalance*: the contrast between the dots and the background was halved for the back surface of the globe compared to the front surface of the globe; 2) *size imbalance*: the size of the dots varied with virtual depth (between 0.051° and 0.198° in diameter, smaller dots on the back surface). These manipulations reliably disambiguated the rotation direction of the globe, as was confirmed by the responses of the participants. The experiment consisted of the two described unambiguous conditions, one ambiguous condition and a baseline condition. The participants completed 3 or 4 trials per condition (3.6 trials on average) of which 3.5% was excluded from the analysis. Inclusion criteria were those described for Experiment 1 and, additionally, perception of the unambiguous globes should stabilize into the percept intended by the disambiguation. All other characteristics of Experiment 2 were the same as those of Experiment 1.

### Experiment 3: Intermittent and continuous pre-exposure

**Figure 4:**
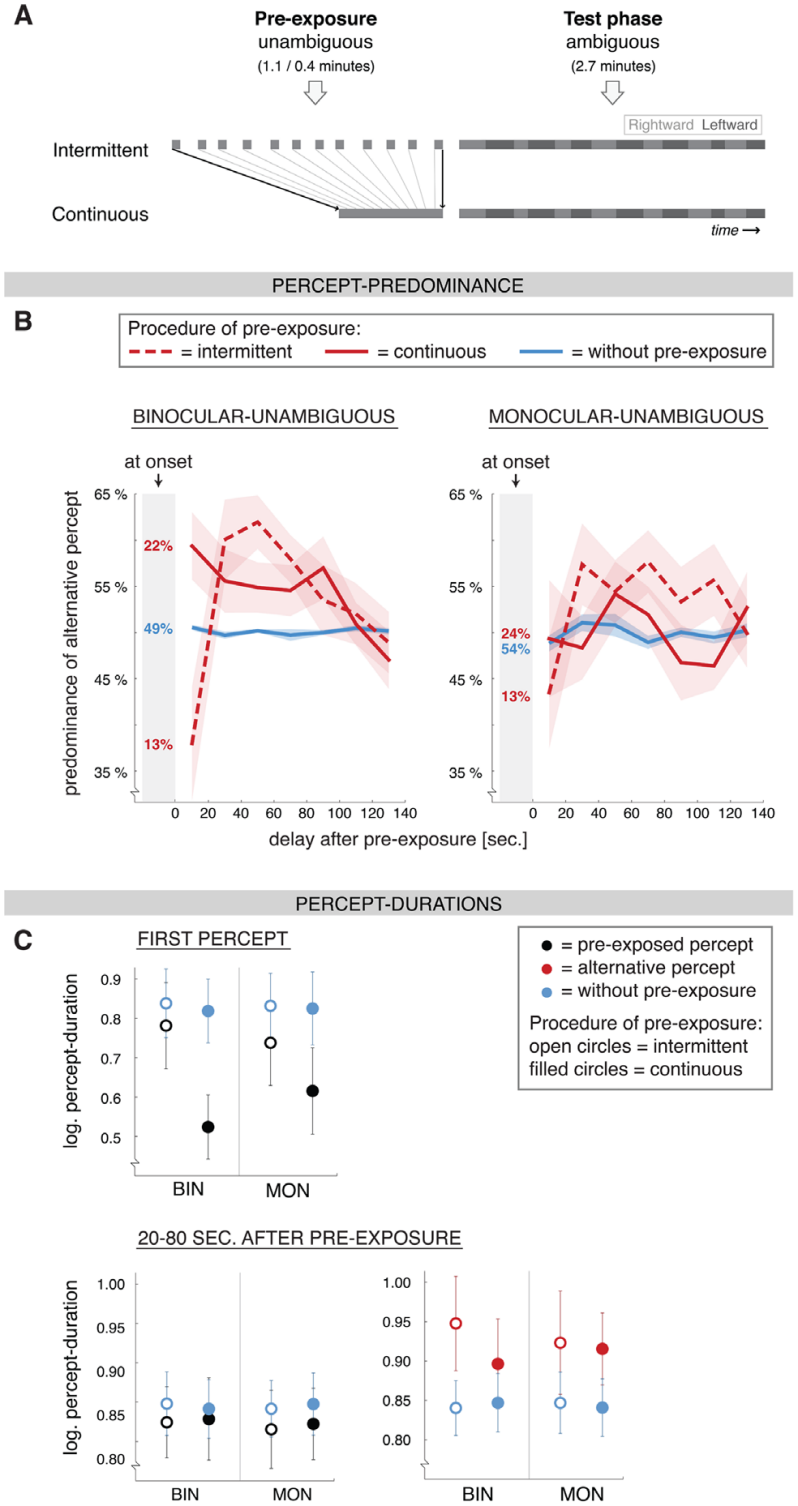
Paradigm and results of Experiment 3: Intermittent and continuous pre-exposure. **A**) We test the influence of the blank periods during the intermittent pre-exposure we compared intermittent pre-exposure with continuous pre-exposure. Both pre-exposure procedures included the same total amount of exposure to the stimulus (i.e. 0.4 minutes). To ensure stable perception during the continuous pre-exposure we used unambiguous stimuli. **B**) The predominance of the alternative percept at the onset of the test phase (number in grey shading) and during subsequent ongoing rivalry (±SEM; bin-width: 20 sec.) for the binocular-unambiguous (*left* graph) and monocular-unambiguous (*right* graph) pre-exposure stimulus. Averaged baseline measure in *blue* (without-pre-exposure). For both stimuli the predominance was larger after the intermittent procedure (*dashed red* lines) than after the continuous procedure (*solid red* lines) in a time-window ranging 20–80 seconds after pre-exposure. In the first time-bin (0–20 sec.) the reverse was true, mainly because the first pre-exposed percept lasted shorter after continuous pre-exposure than after the intermittent pre-exposure (see upper graph in fig. 4C). **C**) *Top row:* The average duration (±SEM) of the first percept in trials that started with the pre-exposed percept (*black*) and trials without pre-exposure (*blue*). For the binocular-unambiguous (BIN) as well as the monocular-unambiguous (MON) stimulus the duration of the first percept was reduced after continuous pre-exposure and not after intermittent pre-exposure. *Bottom row:* The average duration (±SEM) of percepts that occurred between 20 to 80 seconds after pre-exposure (pre-exposed percept in *black*, left graph; alternative percept in *red*, right graph; no pre-exposure in *blue*). The duration of the alternative percept was increased, whereas the duration of the pre-exposed percept was not. Abbreviations: BIN = binocular-unambiguous, MON = monocular-unambiguous.

The pre-exposure phase of each trial consisted of either intermittent presentation, like in Experiments 1 and 2, or continuous presentation. The total presentation duration of the globe was the same for the intermittent and the continuous pre-exposure procedure, i.e. the sum of all short presentations during the intermittent procedure (which took 64 seconds/1.1 minutes, including the blanks) equaled the duration of one long presentation (of 27 seconds/0.45 minutes) during the continuous procedure (fig. 4A). As stabilization cannot be achieved with continuous presentation of the ambiguous stimulus, only the monocular-unambiguous or binocular-unambiguous globes (as described for Experiment 2) were used in the pre-exposure phases of this experiment. There were four experimental conditions (disambiguation method *x* stabilization procedure) and one baseline condition. The test phase of each trial always contained an ambiguous globe and took 2.7 minutes. From experiment 1 we knew that the effect of pre-exposure is smaller when the duration of pre-exposure is smaller. In anticipation of the smaller effect size we used more subjects and more trials. Most participants completed 8 trials per condition (occasionally less, 7.9 trials on average). Based on the inclusion criteria described above 7.0% of the trials was excluded from the analysis.

### Experiment 4: Pre-exposure in binocular rivalry

**Figure 5:**
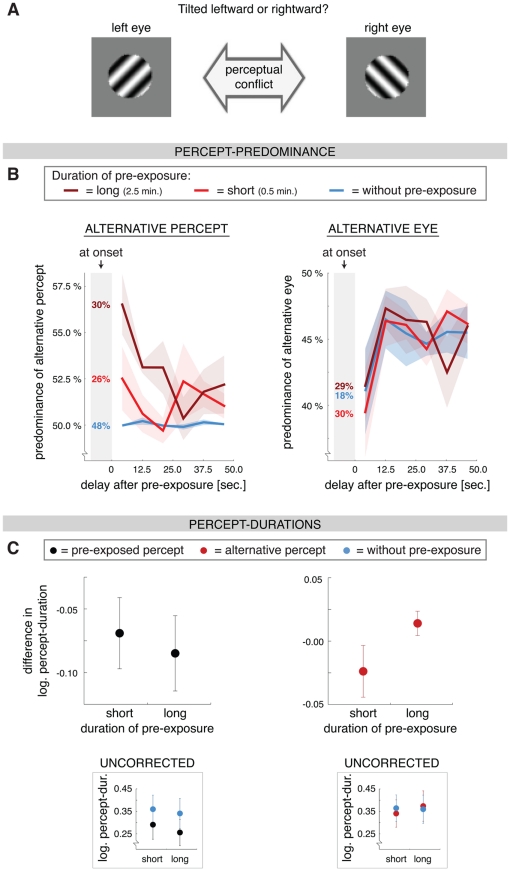
Stimulus and results of Experiment 4 ‘Pre-exposure in binocular rivalry’. **A**) We investigated the perception of binocular gratings to test whether the effects of pre-exposure reflect a general phenomenon among ambiguous stimuli, or whether they are specific to the rotating globe. When a leftward and a rightward tilted grating pattern are presented to the two eyes observers perceive them alternating for several seconds at a time. We used the paradigm presented in figure 1B, with the intermittent viewing period lasting either 0.5 or 2.5 minutes and the test period lasting 50 seconds. In 50% of the trials the grating stimuli were swapped between the eyes at the beginning of the test phase (compared to the intermittent phase of that trial), to be able to dissociate the effects of percept-stabilization from those of eye-stabilization. **B**) The predominance of the alternative percept (*left* graph) and the ‘alternative eye’, i.e. the eye that was suppressed during the pre-exposure (*right* graph) at the onset of the test phase (numbers in grey shading) and during subsequent ongoing rivalry (±SEM; bin-width: 8.3 sec.). In line with the previous experiments in which we used the rotating globe (see fig. 2E), the predominance of the alternative percept during ongoing rivalry was increased after pre-exposure (*red*) compared with a condition without pre-exposure (*blue*). This increase was larger after long pre-exposure (2.5 minutes; *dark red*) than after short pre-exposure (0.5 minutes; *light red*). Rivalry at onset was not influenced by the duration of the pre-exposure. Pre-exposure did not affect the predominance of the alternative eye. In all conditions the predominance of the alternative eye was low initially and near 50% later on. **C**) Lower two graphs: Average duration (±SEM) of percepts that occurred between 0 to 16.7 seconds after pre-exposure (pre-exposed percept in *black*, left graph; alternative percept in *red*, right graph; without pre-exposure in *blue*). Upper two graphs: Same data, but now showing the average *difference in percept duration* between the conditions with and without pre-exposure. The effect of pre-exposure duration is better viewed with this correction, because the variability between the participants in the overall mean percept duration was rather large. The decrease of the duration of the pre-exposed percept is not influenced by the duration of the pre-exposure, whereas the duration of the alternative percept is longer after long pre-exposure than after short pre-exposure (in line with the result for the rotating globe, see fig. 2C).

In this experiment we tested the effect of pre-exposure on two orthogonal black-and-white grating patterns, each grating presented to one eye. When two conflicting images are presented to the two eyes observers perceive only one of them at any given time [29]. We used sine-wave gratings of 1.95 cycles per degree that were titled 45 degrees from vertical to either the left or right and subtended a circular patch of 1.4° in diameter. Participants were instructed to fixate on the centre of the patch (fig. 5A). To enable proper alignment of the eyes a binocular pattern of lines was presented in the periphery of the stimulus. For the individual participants the stimulus and blank durations during intermittent viewing were based on psychophysical pilot-tests (to ensure perceptual stabilization) and averaged to 625 ms and 1581 ms, respectively. The very first intermittent stimulus presentation lasted 8000 ms in all participants, because pilot work showed this reduced the occurrence of mixture percepts (piecemeal combinations of both gratings). There was a baseline condition and two experimental conditions with a pre-exposure duration of either 30 or 150 seconds (which is 0.5 or 2.5 minutes, respectively). The test phase of each trial lasted 50 seconds. Per trial it was randomly determined which grating (leftward or rightward tilted) was presented to which eye. Additionally, in 50% of the trials in the experimental conditions the grating stimuli were swapped between the eyes in the test phase compared to the pre-exposure phase. In this way the grating corresponding to the stabilized *percept* was either in the stabilized *eye* or in the other eye during the second phase. As a consequence, averaging the trials with and without a swap yielded the effect of *percept*-stabilization per se, without any effect of *eye*-stabilization. All other characteristics of Experiment 4 were the same as those of Experiment 1. The participants usually completed 6 trials per parameter-settings, which amounts to 24 trials per condition (occasionally less were completed, 23.4 on average), since there were 4 parameter-settings (being: all combinations of swap/nonswap and leftward/rightward grating in left eye). Based on the criteria described above 4.3% of the trials was excluded from the analysis.

### Analysis of percept durations

The durations of the percepts were derived from the recorded button presses and, considering the generally skewed distribution of percept-durations, were log-transformed (logarithm to base 10) before averaging to avoid a disproportionate contribution of excessively long percepts. Idiosyncratic (subject-specific) bias in the occurrence of the leftward and rightward percepts was taken into account by calculating a weighted average of the data from the baseline condition (without pre-exposure). The purpose of the weighing was to make sure that each percept (leftward or rightward) is counted as ‘pre-exposed’ equally often in the baseline condition and the pre-exposed conditions, so that the idiosyncratic bias between the ‘pre-exposed’ and ‘alternative’ percept, if any, was visible in the baseline condition. For example, if in 75% of the trials with pre-exposure (3 out of 4) the leftward percept was stabilized/pre-exposed during the intermittent phase, the weights of the leftward and rightward percept of the baseline condition were 0.75 and 0.25, respectively. The baseline measure was calculated per percept and per condition (and per eye for the grating stimulus in Experiment 4), for each participant individually. Statistical testing was done using a Greenhouse-Geisser corrected repeated-measures analysis of variance (ANOVA) (unless indicated otherwise). For all tests a two-tailed α of 0.05 was adopted.

### Analysis of percept predominance

The predominance of the alternative percept within a given time-window was calculated as the total time spent seeing the alternative percept divided by the total time perceiving any percept ( = alternative/(pre-exposed+alternative) ). Periods in which neither of the two response buttons were pressed were thus excluded from the analysis. The statistical testing and definition of the baseline measure were the same as for the percept durations.

## Results

The present study was designed to test whether pre-exposure to a perceptually stabilized ambiguous stimulus modifies the perception of ongoing ambiguity in visual information (fig. 1). We attempted to build-up perceptual experience for one of two interpretations of an ambiguously rotating globe (rightward or leftward rotation) by interleaving short presentations with blank periods, which stabilized the perception of the globe. Only trials with proper stabilization (see methods for definition) were included in further analysis of the data (being 97.1%, 93.8%, 96.6% and 93.9% of the trials in Experiments 1, 2, 3 and 4, respectively). To preview our main result: during subsequent continuous viewing of the ambiguous globe the durations of the pre-exposed percept were comparable to a situation without pre-exposure, whereas the durations of the alternative percept were much increased.

### Experiment 1: Ambiguous pre-exposure

**Figure 6:**
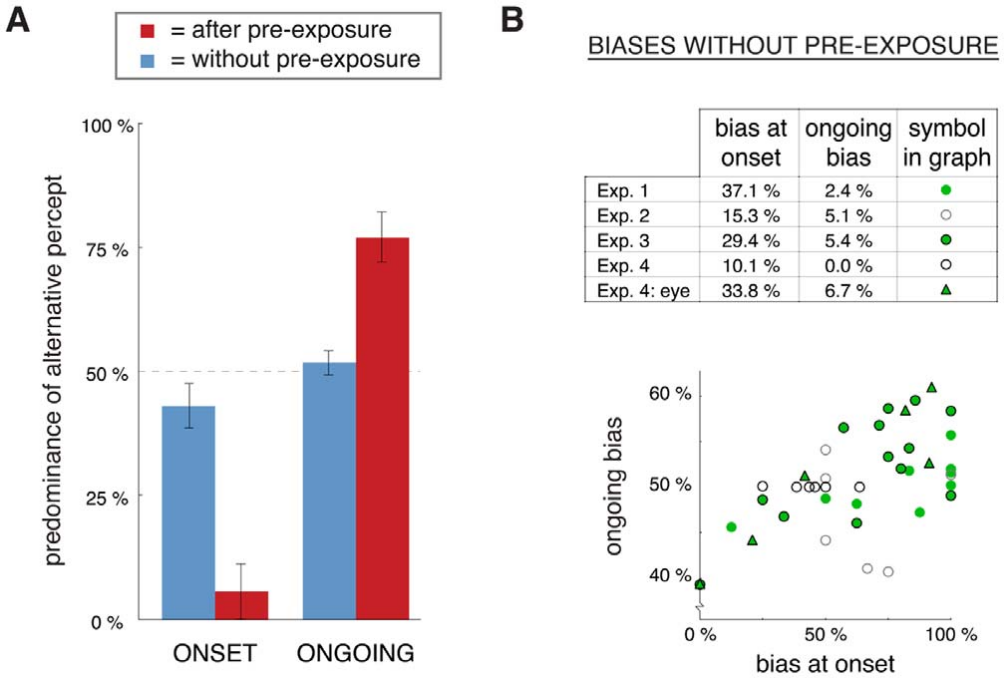
Ongoing rivalry compared with rivalry at onset. **A**) In all four experiments we found an opposite influence of pre-exposure on rivalry at onset and ongoing rivalry (fig. 2A, 3B, 4B and 5B). At onset the alternative percept is suppressed (i.e. there is perceptual stabilization), whereas during ongoing rivalry the alternative percept is facilitated. As an illustration, the graph shows data from Experiment 2, condition with 4.3 minutes of ambiguous pre-exposure (see also fig. 3B). **B**) Idiosyncratic perceptual biases in the baseline condition without pre-exposure, given as the percentage that the rightward percept is seen, or, concerning the eye bias in Experiment 4, the right eye is used. The table presents the mean difference from 50% of the individual biases (i.e. a value of 10% in the table refers to a bias of either 40% or 60%). Biases were high at onset and very small during ongoing rivalry. The graph shows the bias at onset and during ongoing rivalry for the individual participants in all four experiments. The ongoing biases are all small (ranging from 39% to 61%), but correlated positively with the bias at onset (which ranged from 0% to 100%) for Experiment 1, Experiment 3 and the eye bias in Experiment 4 (indicated with green symbols).

We varied the amount of pre-exposure by changing the duration of the intermittent period. During this period the same percept was seen repeatedly at almost all of the presentations of the stimulus (97.5%, 99.4%, 97.6%, 97.7% and 97.2% of the presentations for the extra short to extra long pre-exposures, respectively). Regardless of the duration of the pre-exposure the tendency to perceive the pre-exposed percept at the onset of the test phase was stronger after pre-exposure (96.7%, 95.0%, 100%, 85.8% and 91.7% of trials for the extra short to extra long pre-exposure durations, respectively) than without pre-exposure (79.2%, this baseline value is also relatively high due to relatively large idiosyncratic biases, fig. 6B).

The predominance of the alternative percept during the test phase was calculated in successive time-bins with a width of 30 seconds (fig. 2A). The predominance was defined as the percentage of time that the percept was seen within the time-bin. From 0.5 till 3.5 minutes after extra long pre-exposure (4.3 minutes) the predominance of the alternative percept was significantly larger than the baseline measure (time-bins 2-5 and 7: all t>3.0, all p<0.05; time-bin 6 was marginally significant: t = 2.2, p = 0.06). During this time-window the increase in predominance of the alternative percept was visible in the data of every individual participant that we tested (fig. 2A, right graph). The effect of pre-exposure was not significant in the first time-bin, presumably because of the first percept at the onset of the test-phase was almost invariably the one seen during intermittent presentation.

After long pre-exposure (2.2 minutes) the predominance of the alternative percept was increased in a time-window ranging from 0.5 till 2.5 minutes (time-bins 2–5: all t>2.8, all p<0.05) and after medium-length pre-exposure (1.1 minutes) this was true for a time-window spanning 0.5 till 1.0 minutes (time-bin 2: t = 2.5, p<0.05). The effect of pre-exposure thus lasted longer when the pre-exposure itself took longer (1.0, 2.5 and 3.5 minutes after a pre-exposure of 1.1, 2.2 and 4.3 minutes, respectively; fig. 2A, left graph). Additionally, the magnitude of the effect depended on the duration of the pre-exposure. In a time-window ranging from 0 till 2.5 minutes after pre-exposure (all *F*>4.3, all p<0.05) the longer pre-exposure durations resulted in a larger predominance of the alternative percept and this trend was also visible in a time-window ranging from 2.5 till 4 minutes after pre-exposure (all *F*>3.4, all p≤0.08). Thus, the pre-exposed percept was reliably seen at the onset of the test phase for all pre-exposure durations, whereas the alternative percept predominated during continuous viewing. The magnitude and duration of the predominance of the alternative percept increased when the duration of pre-exposure was longer (fig. 2A).

To see what the influence of pre-exposure is on the duration of the perceptual epochs we analyzed the individual durations of the pre-exposed and alternative percept after extra-long exposure to intermittent presentation (4.3 minutes; fig. 2B and 2C). To avoid a disproportionate contribution of excessively long percepts we further analyzed the logarithmic transformation of the percept durations. Compared to a condition without pre-exposure, continuous viewing after pre-exposure was characterized by long durations of the alternative percept, whereas the duration of the pre-exposed percept was not much affected. The duration of the alternative percept was longest shortly after pre-exposure and gradually decayed to baseline afterwards (fig. 2B, right graph; 1^st^–5^th^ occurrence of the alternative percept: all t>2.6, all p<0.05). For the pre-exposed percept there was a trend toward a slight decrease in duration compared with baseline (fig. 2B, left graph; only significant for 2^nd^ and 6^th^ occurrence of the pre-exposed percept: both t≤−2.6, both p<0.05; see methods for definition of baseline). In a pilot experiment we used a pre-exposure duration of 2.2 minutes and a test period of 15 minutes and we found no late or cyclic effects of pre-exposure after this initial effect starting early in the test-phase.

To investigate how the modulation of the percept duration depended on the duration of the pre-exposure we compared the average of all percepts ending within 1.5 minutes after pre-exposure across the different pre-exposure durations. Within this time-window the effect of pre-exposure was maximal for all pre-exposure durations (fig. 2A). A 2-way repeated-measures ANOVA over *pre-exposure duration* and *condition* (i.e. with/without pre-exposure) revealed that the decrease in the duration of the pre-exposed percept was not significant (*F*_(1, 9)_ = 3.3, p = 0.1; fig. 2C). Also, the duration of the pre-exposed percept was not modulated by the duration of the pre-exposure (main effect and interaction effect were not significant: both *F*≤0.7, both p≥0.6). A least-squares repeated-measures regression confirmed that the duration of the pre-exposed percept was not influenced by the duration of the pre-exposure (fig. 2C, left graph; slope = −0.02, t = −0.4, p = 0.3; slope for baseline measure: −0.02, t = −1.0, p = 0.2).

For the duration of the alternative percept, on the other hand, a 2-way repeated-measures ANOVA revealed a significant interaction effect (*F*_(2.4, 21.9)_ = 4.6, p<0.05; fig. 2C, right graph). Further testing showed that the duration of the alternative percept changed with the duration of the pre-exposure (*F*_(2.1, 19.1)_ = 6.1, p< = 0.01), while the baseline measure did not (*F*_(1.4, 12.7)_ = 0.8, p = 0.4). A repeated-measures regression indicated that the duration of the alternative percept increased with the duration of the pre-exposure in a near-linear fashion (slope = −0.23, t = 5.4, p<0.001; slope for baseline measure: 0.02, t = 1.0, p = 0.2). There was thus no evidence of saturation of the effect with longer durations of pre-exposure.

Taken together, the results of Experiment 1 revealed a functional link between perceptual stabilization of an intermittently presented ambiguous stimulus and later continuous viewing of the same stimulus: the percept that was suppressed during intermittent viewing (i.e. rivalry at onset) predominated during continuous viewing. The time-span and strength of the effect on ongoing rivalry (but not the effect on rivalry at onset) depended on the amount of prior exposure to the perceptually stabilized stimulus (fig. 2).

### Experiment 2: Unambiguous pre-exposure

The effects of pre-exposure found in Experiment 1 could reflect an influence of previous perceptual state, i.e. the content of perceptual awareness, or they could be specifically related to perceptual decision-processes under conditions of visual ambiguity. To differentiate these two factors we compared ambiguous pre-exposure with unambiguous pre-exposure. In this latter condition the perceptual state is the same, but it is determined by exogenous stimulus manipulations as opposed to endogenous decision-making mechanisms. We used an ambiguous stimulus in all test phases, but in the intermittent pre-exposure phase we presented either one of three stimuli: an ambiguous globe, a globe disambiguated with disparity (‘binocular-unambiguous’) or a globe disambiguated with a contrast- and size-imbalance (‘monocular-unambiguous’) (fig. 3A). These three cases were similar with respect to the stabilization of perception during pre-exposure (99.2%, 99.8% and 99.5% of the presentations, respectively), as well as the tendency to perceive the pre-exposed percept at the onset of the test phase (94.4%, 86.1%, 95.8%, respectively, compared with 51.9% in the condition without pre-exposure).

After ambiguous as well as unambiguous pre-exposure the predominance of the alternative percept (calculated per 30 seconds) was increased compared with the baseline condition without pre-exposure (fig. 3B). The time-span of this effect overlapped between the three different pre-exposure stimuli (Ambiguous ⇒ time-bins 3–6: all t>3.1, all p<0.05; Binocular-unambiguous ⇒ time-bins 2–4 and 7: all t>3.3, all p<0.05; time-bins 5–6 were marginally significant; Monocular-unambiguous ⇒ time-bins 3, 8 and 9: all t>2.6, all p<0.05; time-bins 5–7 were marginally significant; time-bin 14 showed significant decrease: t = −9.5, p<0.001).

From 0.5 till 4.5 minutes after pre-exposure (time-bins 2–9) there were significant increases in the predominance of the alternative percept for at least one of the three pre-exposure stimuli. We performed a repeated-measures ANOVA over this time-window and found a significant main effect of the pre-exposure stimulus (*F*_(1.7, 8.6)_ = 5.5, p<0.05; the time-bins did not differ from each other in this respect). Partial testing revealed that the increase in predominance of the alternative percept was significantly larger after ambiguous pre-exposure than after monocular-unambiguous pre-exposure (*F*_(1, 5)_ = 15.6, p<0.05). The binocular-unambiguous case was an intermediate, as it did not significantly differ from either of the other two stimuli (both *F*_(1, 5)_≤3.7, both p>0.1; there were no effects of time-bin in the partial tests). The difference between monocular- and binocular-unambiguous pre-exposure was further explored in Experiment 3 and did reach statistical significance there.

The log-transformed duration of the alternative percept showed the same pattern of results. As in Experiment 1, the duration of the pre-exposed percept was slightly decreased after pre-exposure (*F*_(1, 5)_ = 8.1, p<0.05), but was not influenced by the type of stimulus that was pre-exposed (*F*_(1.8, 8.7)_ = 0.7, p = 0.5). The duration of the alternative percept, on the other hand, differed for the different pre-exposure stimuli (*F*_(1.5, 7.7)_ = 4.9, p<0.05) and was significantly longer after ambiguous compared with monocular-unambiguous pre-exposure (*F*_(1, 5)_ = 9.4, p<0.05; fig. 3C). The baseline measures also did not differ between the 3 conditions (*F*_(1.1, 5.3)_ = 0.2, both p = 0.7).

In overview, the effect of pre-exposure was qualitatively the same for the ambiguous and unambiguous cases. However, monocular-unambiguous pre-exposure had a smaller influence on ongoing rivalry than ambiguous pre-exposure, both in terms of the predominance of the alternative percept and the durations of the alternative percept. Binocular pre-exposure showed intermediate values. Such a difference between the pre-exposure stimuli was not observed for rivalry at onset.

### Experiment 3: Intermittent and continuous pre-exposure

In this experiment we introduced a continuous pre-exposure procedure that consisted of a single continuous presentation of the globe and compared this with an intermittent pre-exposure paradigm. The blank periods in an intermittent procedure may allow the system to partially return to baseline, thereby attenuating the effect of pre-exposure. Alternatively, by forcing the visual system to repeatedly make perceptual decisions at each stimulus onset, effect of pre-exposure may be stronger after intermittent than after continuous pre-exposure. Importantly, we kept the total duration of exposure to the stimulus equal for both paradigms (i.e. 0.45 minutes; fig. 4A). Considering that ambiguous and unambiguous pre-exposure have qualitatively similar effects (see Experiment 2) we used unambiguous globes in all pre-exposure periods, as these ensured stable perception during the continuous as well as the intermittent pre-exposure. The percentage of time that the same percept was seen during pre-exposure was 99.5% and 99.7% in the intermittent and continuous binocular-unambiguous conditions, and 99.9% and 98.8% in the intermittent and continuous monocular-unambiguous conditions, respectively.

Whereas rivalry at onset was not influenced by the duration of pre-exposure in Experiment 1 or the pre-exposure stimulus in Experiment 2, we did find an effect of the pre-exposure procedure on rivalry at onset in Experiment 3. After continuous pre-exposure the first percept was shorter than the baseline measure (*F*_(1, 12)_ = 6.1, p<0.05), whereas this was not the case after intermittent pre-exposure (fig. 4C, top row; *F*_(1, 12)_ = 0.5, p<0.5; difference from baseline *x* pre-exposure procedure: *F*_(1, 12)_ = 15.3, p<0.01). In trials where the test phase started with the alternative percept instead of the pre-exposed percept the duration of the first percept was not influenced by pre-exposure procedure (*F*_(1, 12)_ = 0.9, p = 0.4). Only the duration of the first pre-exposed percept was influenced. The intermittent and continuous procedure did not differ much in the percentage of trials in which the test phase started with the pre-exposed percept (87% and 77%, respectively; small difference may be related to shorter percepts being harder to track with button presses).

From 0 till 80 seconds after pre-exposure (time-bins 1–4) there were significant changes in the predominance of the alternative percept (compare with the baseline measure) for at least one of the four conditions (fig. 4B). We performed a repeated-measures ANOVA over this time-window and found that the effect of the pre-exposure procedure was different in the first time-bin after pre-exposure ( = first 20 seconds) compared with 20–80 seconds after pre-exposure (time-bin 2–4), reflecting the difference in rivalry at onset between the procedures (described above). Between 20 and 80 seconds after pre-exposure the predominance of the alternative percept was larger after intermittent pre-exposure than after continuous pre-exposure (main effect of *procedure*: *F*_(1, 12)_ = 9.1, p<0.05; fig. 4B). Also, the predominance of the alternative percept was larger when the binocular-unambiguous stimulus was used than when the monocular-unambiguous pre-exposure stimulus was used (main effect of *pre-exposure stimulus*: *F*_(1, 12)_ = 7.9, p<0.05; in line with Experiment 2). Regarding the percept durations, the duration of the alternative percept was increased compared with the baseline measure (*F*_(1, 12)_ = 4.8, p<0.05; fig. 4C, bottom row), but the effect of pre-exposure procedure was not significant (*F*_(1, 12)_ = 0.9, p = 0.4). The duration of the pre-exposed percept did not differ from the baseline measure (*F*_(1, 12)_ = 0.02, p = 0.9; first percept of test-phase excluded from analysis).

To summarize, the effect of pre-exposure on ongoing rivalry was qualitatively the same, but smaller when the pre-exposure consisted of one continuous presentation (continuous procedure) compared with a situation where blanks were included in the pre-exposure phase (intermittent procedure). In line with Experiments 1 and 2 the duration of alternative percept was increased, whereas the duration of the pre-exposed percept was not affected. Additionally, the first occurrence of the pre-exposed (but not the alternative) percept after continuous pre-exposure was shorter in duration than during the condition without pre-exposure, whereas this was not the case after intermittent pre-exposure.

### Experiment 4: Pre-exposure in binocular rivalry

To see whether the effect of pre-exposure is specific for the rotating globe, or whether it extends to other ambiguous stimuli, we also tested binocular rivalry (orthogonal gratings; fig. 5A). During binocular rivalry we can identify a pre-exposed percept, but also a ‘pre-exposure eye’, i.e. the eye that was presented with the pre-exposed percept used during pre-exposure. To be able to dissociate the effects of the percept of pre-exposure from the eye of pre-exposure we switched the grating patterns between the eyes in half of the trials as soon as the test phase had ended. As with the rotating globe, perception was stabilized during the intermittent pre-exposure phase (the percentage of presentations with the same percept was 97.2% and 97.7% for the short and long pre-exposure duration, respectively).

During ongoing rivalry the predominance of the *eyes* was not influenced by pre-exposure (difference from condition without pre-exposure: all t>1.9, all p>0.1; overall ANOVA: *F*_(1, 5)_ = 0.1, p = 0.7). We calculated the predominance over 6 equally sized time-bins (test phase lasted 50 sec., bin-width was 8.3 sec.). In both pre-exposure conditions there was a strong tendency to see the grating in the ‘pre-exposure eye’ at the start of the test phase (in 69.6% and 71.3% of the trials for the short and long pre-exposure durations, respectively; fig. 5B, right graph). The same eye was also predominant at the start of the trials without pre-exposure (i.e. in 82.2% of the trials). This was due to idiosyncratic eye-biases, i.e. most subjects tend to see the image presented to one specific eye at the beginning of any trial. This eye becomes the ‘pre-exposure eye’ in the conditions with pre-exposure and it is also the eye that is initially used in the condition without pre-exposure. The numbers suggest that this eye-bias was slightly reduced at onset of the test-phase after pre-exposure (from 82.2% to about 70%), but this difference was not significant (*F*_(1, 5)_ = 0.1, p = 0.7; fig. 5B right graph). Regarding the *perceptual* bias (i.e. pattern bias), there was a significant effect of pre-exposure on rivalry at onset, indicating that perceptual stabilization occurred (*F*_(1, 5)_ = 7.6, p<0.05; fig. 5B left graph).

In all experiments with the rotating globe we found an opposite influence of pre-exposure on rivalry at onset and ongoing rivalry (fig. 2A, 3B, 4B). At onset the alternative percept is suppressed (i.e. there is perceptual stabilization), whereas during ongoing rivalry the alternative percept is facilitated. In line with this, the predominance of the alternative percept was also increased during *ongoing* binocular rivalry after pre-exposure compared with the condition without pre-exposure (fig. 5B, left graph). After long pre-exposure this increase in predominance was significant within a delay of 0 to 16.8 seconds (time-bins 1–2: both t>3.9, both p<0.05). In this time-window this effect of pre-exposure was stronger after long pre-exposure than after short pre-exposure (*F*_(1, 5)_ = 7.8, p<0.05). We also analyzed the average duration of the percepts that occurred between 0 and 16.8 seconds after pre-exposure. The pre-exposed and alternative percept were differentially influenced by pre-exposure (*F*_(1, 5)_ = 8.3, p<0.05). Based on the results for the rotating globe we expected the duration of the alternative percept to be longer after long pre-exposure than after short pre-exposure. There was indeed a trend toward this difference (*F*_(1, 5)_ = 4.4, p = 0.09; compare fig. 5C to fig. 2C), but the overall increase was not significant (*F*_(1, 5)_ = 0.1, p = 0.8). The duration of the pre-exposed percept was decreased compared with the condition without pre-exposure (*F*_(1, 5)_ = 7.0, p<0.05), but was not influenced by the duration of the pre-exposure (*F*_(1, 5)_ = 0.4, p = 0.5).

In comparison with the rotating globe, the effect of pre-exposure was qualitatively the same for binocular rivalry. The pre-exposed percept was initially seen at the onset of the test phase, whereas the alternative percept predominated during subsequent ongoing rivalry. The duration of the alternative percept during ongoing rivalry was longer when the pre-exposure had lasted longer. The duration of the pre-exposed percept, on the other hand, was decreased during ongoing rivalry following pre-exposure, but this decrease was not influenced by the duration of the pre-exposure. The effect for binocular rivalry appeared to be smaller in size and less long-lasting than the effect for the rotating globe (compare fig. 5B to fig. 2D). Pre-exposure did not affect the predominance of the eye that was dominant during pre-exposure.

### Idiosyncratic perceptual bias

In the absence of bias the predominance of both the rightward and the leftward percept would be 50%. However, we found that idiosyncratic biases were present in the condition without pre-exposure in all of the experiments. Interestingly, the biases were much more extreme at the onset of rivalry (initial percept) than during ongoing rivalry. In Experiment 1, for example, there was on average a 37% distance from 50% in the predominance of the rightward percept at onset of the condition without pre-exposure, whereas this distance was only 2.4% during ongoing rivalry without pre-exposure. A similar pattern was found for the other experiments (fig. 6B). Although the ongoing biases were small, there was a significant positive correlation between onset bias and ongoing bias for Experiment 1 (regression coefficient = 0.07, t = 3.2, p<0.05), Experiment 3 (regression coefficient = 0.15, t = 3.4, p<0.01) and the eye bias in Experiment 4 (regression coefficient = 0.19, t = 4.8, p<0.01). The presence of these biases stresses the importance of the weighted baseline measure used in the analyses described above (see methods), which ruled out any contribution of idiosyncratic bias to the effects of pre-exposure.

## Discussion

We investigated ongoing conscious perception of ambiguous visual information after observers were pre-exposed to a perceptually stabilized sequence of the same stimulus. The subsequent ongoing rivalry between the possible interpretations of the ambiguous stimulus was biased toward the alternative percept, i.e. the percept that was suppressed during the pre-exposure. In contrast, the initial interpretation of the stimulus showed a bias toward pre-exposed instead of the alternative percept (fig. 6A; perceptual stabilization, see [10]–[12]). Rivalry at onset thus had a different dependence on pre-exposure than ongoing perceptual rivalry. Furthermore, the bias at onset was only reflected in the perceptual choice and not in the duration of the first percept, whereas a modification of percept durations was responsible for the bias toward the alternative percept during ongoing rivalry. Previous research has indicated that rivalry at onset and ongoing rivalry also differ in the dynamics of the perceptual choices [5], [7], the influence of idiosyncratic perceptual biases [6] and the influence of attention [8], [9], [16]. Taken together, our results reaffirm the difference between gaining dominance at the onset of an ambiguous stimulus and *re*gaining dominance during ongoing rivalry.

The facilitative effect of pre-exposure at the onset of an ambiguous stimulus is not overwritten by unrelated intervening stimulus-presentations [17], [22], [33] and can be influenced by complex task-characteristics [16], [34]. It may be mediated by a greater neural sensitivity to the pre-exposed percept, i.e. a greater ‘readiness to respond’. For example, a change in sensitivity, rather than a change in *activity*, is particularly suited to produce a steeper upstroke in the neural activity for that percept at the onset of the stimulus [5], [35]. A change in sensitivity may not be very effective during ongoing rivalry, because there is already neural activity for both percepts, albeit sub-threshold for the suppressed percept [35], [36]. In this situation the mutual inhibition between the neurons or the saturation level of the neural activity are more likely candidates for mediating the effect of pre-exposure. Specifically, we had hypothesized either a fatigue-like decrease in the duration of the pre-exposed percept during ongoing rivalry (suppression) or a ‘memory-like’ increase (facilitation). For the alternative percept we initially expected no effect, but considering Levelt's second proposition of binocular rivalry the effect of pre-exposure may also transfer to the perceptual durations of the alternative percept [29], [30] (see hypotheses in fig. 2B).

Our results indicate that the duration of the alternative percept was increased after pre-exposure, while the duration of the pre-exposed percept remained largely unaffected (fig. 2C), which supports a Leveltian transfer of the suppression hypothesis. It is not likely that this is caused by fatigue in the neurons coding for the pre-exposed percept, since that would logically lead to shorter durations of the pre-exposed percept. Nonetheless, considering that the effect of pre-exposure is specific for retinotopic location [37], [38], it seems reasonable that sensory neurons tuned to the feature-differences between the percepts are involved. A role of sensory brain regions is further supported by a recent transcranial magnetic stimulation study [39] and several models of perceptual rivalry [5], [35]. Therefore, we speculate that the neurons coding for the alternative percept may have been primed – without being fatigued - as a consequence of subthreshold activation during pre-exposure [36]. An alternative, but not mutually exclusive, suggestion is that the cross-inhibition between the neurons coding for the two percepts is affected by the pre-exposure, rather than the activity in the neurons themselves. Long-term adaptation in this inhibitory mechanism has been reported recently for binocular rivalry [40].

Pre-exposure also had a nonspecific, i.e. percept-invariant, effect on all percept-durations. After pre-exposure the durations were slightly shorter compared with a situation without pre-exposure (fig. 2C), in line with the increase in the perceptual alternation-rate reported previously [13], [14]. The duration of the pre-exposed percept was affected only by this small and nonspecific effect, which reached significance just in 2 out of our 4 experiments. Our results suggest that for the alternative percept the nonspecific decrease in duration is masked by a facilitative effect (increase in duration) that becomes increasingly larger with longer pre-exposure. This interpretation would explain why there was a slight decrease in duration with very short (≤30 seconds) durations of the pre-exposure: the decrease outweighed the increase (fig. 2C and 5C). For binocular rivalry as well as ambiguous structure-from-motion the facilitative effect outweighed the nonspecific decrease by far when the pre-exposure lasted longer. Consequently, the predominance of the alternative percept, defined as the percentage of time that this percept was seen, was much larger after pre-exposure than without pre-exposure. There were no signs of saturation or ceiling of this effect when the duration of the pre-exposure was extra long (max. 4.3 minutes in our experiments; fig. 2C), suggesting that the duration of the alternative percept becomes even longer with pre-exposure durations that exceed those measured in the present study.

Not only the magnitude of the effect of pre-exposure (see above), but also its lifetime scaled with the duration of the pre-exposure. The lifetime of the ‘perceptual memory’ was thus proportional to the duration of the relevant perceptual experience (for related findings with onset-rivalry see [12], [17]). Moreover, the prevalence of the alternative percept was surprisingly long lasting (4.5 minutes in Experiment 2, after 4.3 minutes of pre-exposure). Previously reported interdependencies between consecutive percepts during *ongoing rivalry* were short-lived (regarding percept duration: [41]; survival probability: [42]; and percept identity: [43], [44]. For *rivalry at onset* effects of unambiguous pre-exposure have been reported that lasted hours/days [38]. The present results shows that the *ongoing* perception of visual ambiguity is also subjected to longer-term effects of prior perception, at the least on the scale of minutes.

During binocular rivalry (Experiment 4; fig. 5) the predominance of the two eyes was not affected by pre-exposure, while the predominance of the two percepts showed a pattern similar to what was found for the ambiguous rotating globe. This finding is surprising given previous reports that perceptual stabilization during intermittent binocular rivalry is more eye-based than percept-based [45]. However, at the onset of binocular rivalry there is a large influence of idiosyncratic eye-bias and this eye-bias is not affected by pre-exposure (fig. 5B and 6B). The tendency to repeatedly see the image presented to the same eye during intermittent binocular rivalry, even when the images are swapped between the eyes (see [45]), was thus driven by idiosyncratic eye-bias and not by a gain in dominance of that eye. There was even a small (but not significant) decrease in eye-bias after pre-exposure, instead of an increase (fig. 5B). By swapping the images between the eyes at the start of the continuous test phase in 50% of the trials we could average out this eye-based effect and we found that there is a small, but significant, effect of *perceptual* stabilization after pre-exposure (in line with [45]). The pre-exposed percept was more likely to be seen at onset of the stimulus after pre-exposure than without pre-exposure (fig. 5B). During subsequent ongoing binocular rivalry the predominance of the alternative percept was increased, in line with the results for the ambiguous structure-from-motion stimulus. There were almost no idiosyncratic eye-biases during ongoing binocular rivalry (see [6], [46] for related findings), regardless of whether the condition included pre-exposure or not (fig. 5B and 6B).

Our findings were not specific to ambiguous pre-exposure. We found that unambiguous pre-exposure resulted in a qualitatively similar effect. In our paradigm the pre-exposed percept was facilitated at onset of the test phase after ambiguous as well as unambiguous pre-exposure. Suppression of the pre-exposed percept at onset of the stimulus has often been reported after unambiguous pre-exposure [23]–[26], but facilitation is common after long blank intervals [15], [19]–[20]. During ongoing rivalry there was facilitation of the alternative percept after ambiguous as well as unambiguous pre-exposure (see also predominance ratios described in [47]–[49]), indicating that the effect of pre-exposure is not reliant on perceptual decision-processes under conditions of ambiguity. However, the magnitude of the effect was smaller when the pre-exposed stimulus was disambiguated with monocular depth-cues (i.e. a contrast- and size-imbalance) than when it was ambiguous or disambiguated with a binocular depth-cue (disparity). The disparity-defined stimulus also seemed to have a smaller effect than the ambiguous stimulus, but this difference was not significant (fig. 3B and 4B). We interpret this as an indication that the site of neural processing where ambiguous structure-from-motion is resolved has more overlap with the site where disparity information is processed than with the processing-level of basic stimulus features such as size and contrast. During the perception of ambiguous structure-from-motion perceptual decisions are indeed reflected in the activations of brain regions that are sensitive to disparity [50].

One could also suggest that the effect of unambiguous pre-exposure was smaller because unambiguous stimuli lead both to stimulus-based adaptation and percept-based ‘memory’, which counteract each other. However, given that the former is likely to have a suppressive effect on the pre-exposed percept, while the latter facilitates the alternative percept, these effects would strengthen rather than counteract each other in terms of the predominance of the percepts. Also, it is unlikely that these effects would last equally long, considering that the suppressive effect on the pre-exposed percept that we found after continuously (but not intermittently) presented unambiguous pre-exposure lasted for only one perceptual epoch. In other words, it merely influenced rivalry at onset. In line with our results regarding ongoing rivalry, it was previously reported that rivalry at onset is also influenced more strongly by ambiguous than by unambiguous pre-exposure in a specific location-contingent perceptual learning paradigm [38], [51]. Interestingly, using the same paradigm, pre-exposure with a combination of binocular and monocular depth-cues had a stronger effect on rivalry at onset than by pre-exposure with monocular depth-cues only [52].

If the cross-inhibition between the two percepts is indeed modified by pre-exposure, as proposed above, there is an additional explanation of our results regarding unambiguous pre-exposure. One could propose that less inhibition of the suppressed percept is needed when there is more low-level percept-specific information as evidence for the dominant percept. This weaker inhibition *during* pre-exposure might result in a weaker modulation of the inhibition *after* pre-exposure. A similar reasoning might explain why we found a smaller effect of continuously presented unambiguous pre-exposure than intermittently presented unambiguous pre-exposure, even though the total presentation-time was kept constant between the two procedures (fig. 4B). It could be that the repeated onset of stimulation during the intermittent pre-exposure more strongly activated mutual inhibition than the continuous presentation.

### Conclusion

The present data show that previously perceived interpretations dominate at the onset of ambiguous sensory information, whereas alternative perceptual interpretations tend to dominate with prolonged viewing. This effect of previous experience on the perception of ongoing sensory ambiguity can last for several minutes and is larger when the pre-exposure lasted longer. We suggest that the reported effects could be related to priming of the suppressed percept during pre-exposure. Alternatively, learning processes in the mutual inhibition between the possible perceptual interpretations may play a role. The effect was found for perceptual as well as binocular rivalry and was larger after ambiguous pre-stimulation than after unambiguous pre-stimulation. In all, our results are compatible with a mechanism that optimizes performance by learning from experience in the following manner: the nature of new sensory input is assessed quickly through the retention of past experience, while alternative interpretations are considered after continued evaluation of the information.
